# Supplementary figures and images for: Screening of immunosuppressive factors for biomarkers of breast cancer malignancy phenotypes and subtype-specific targeted therapy
Source: PeerJ. 2019 Jun 27;7:e7197. doi: 10.7717/peerj.7197 (PMC6599676; doi:10.7717/peerj.7197)

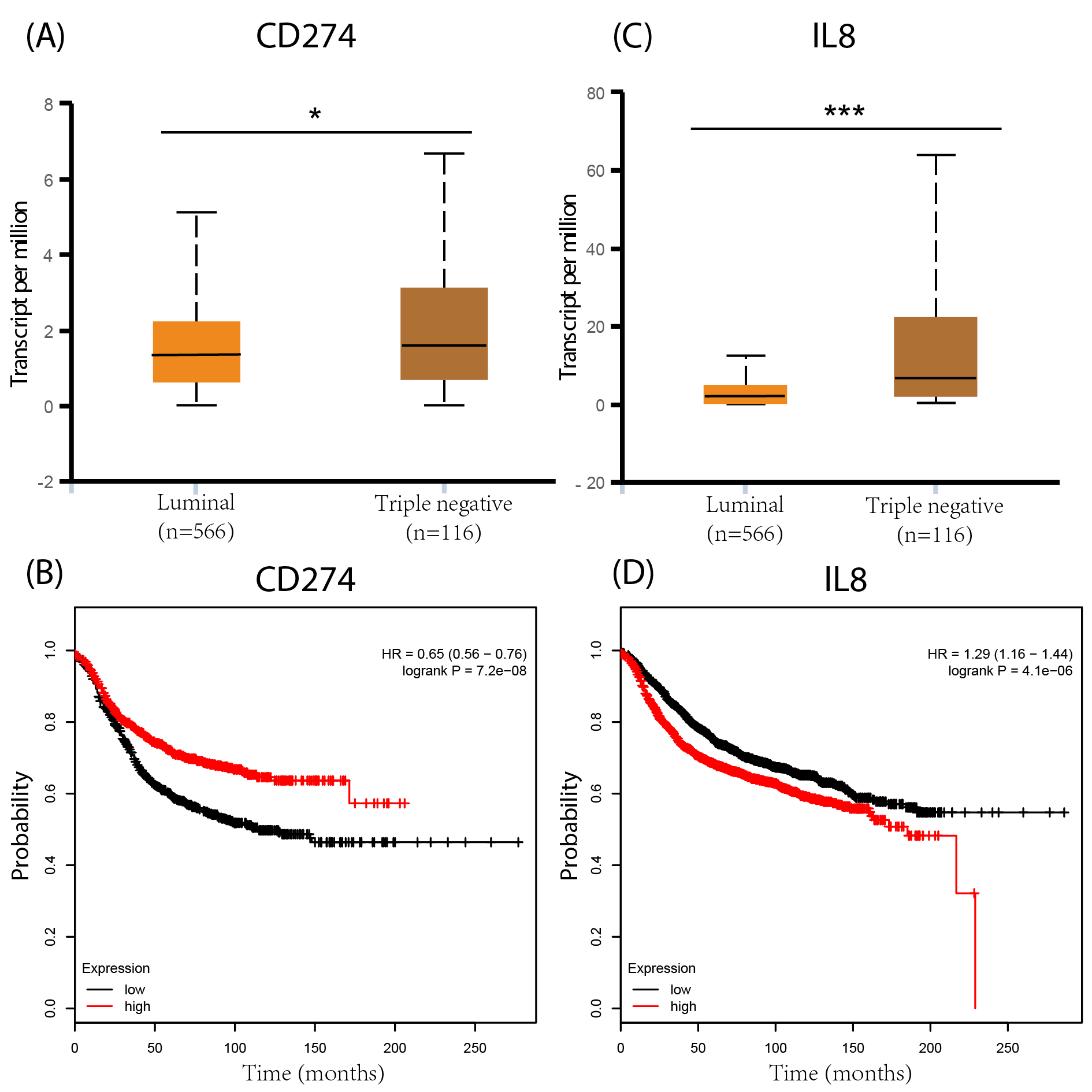

Supplement: Supplemental Information 1 — (A, C) CD274 and IL-8 are highly expressed in Basal-like tumours; (B, D) higher IL-8 expression is associated with poor prognosis, while CD274 is the opposite.. [file peerj-07-7197-s001.png]
